# Patient perspectives on how to improve education on medication side effects: cross-sectional observational study at a rheumatology clinic in The Netherlands

**DOI:** 10.1007/s00296-021-04815-5

**Published:** 2021-03-17

**Authors:** Mirjam C. Hegeman, Jolanda A. Schoemaker-Delsing, Jacqueline T. M. Luttikholt, Harald E. Vonkeman

**Affiliations:** 1 Department of Psychology, Health and Technology, University of Twente, Enschede, The Netherlands; 2 Department Rheumatology and Clinical Immunology, Medisch Spectrum Twente, Enschede, The Netherlands

**Keywords:** Rheumatoid arthritis, Medication education, Medication side effects, Patient perspectives, Shared decision-making

## Abstract

Rheumatoid arthritis (RA) patients often report lacking information on medication side effects. The aims of this study were to observe how rheumatology healthcare providers deliver medication information and to determine in which specific domains information is missing. First, 12 single-blinded structured observations were performed during regular RA patient consultations. The observers noted whether and how medication and medication side effects were discussed. Second, 100 RA patients were asked to fill out an adaptation of the Satisfaction with Information about Medicines Scale (SIMS). Medication was discussed during all observed consultations. With new medication, its purpose and mode of action were explained in all cases, but possible side effects in only 33%. Overall, medication side effects were discussed in 58% of consultations. Most information delivery was verbal (92%). Response rate to the questionnaire was 61%. Overall satisfaction with medication education was mean 7.3 (± 1.9) (NRS 0–10) with a comparable high SIMS total satisfaction sum score of mean 12.3 (± 4.4). At subscale score levels, 89% were satisfied with the amount of information on the action and usage of medication, but only 47% with the information on the potential problems of medication. RA patients express overall high satisfaction with their medication education but there is still an unmet need for information on potential risks and side effects. Using the SIMS questionnaire in daily clinical practice may help focus medication education to the needs of the individual patient.

**Supplementary Information:**

The online version contains supplementary material available at 10.1007/s00296-021-04815-5.

## Introduction

Rheumatoid arthritis (RA) is a chronic systemic autoimmune disease characterized by joint inflammation, leading to progressive joint destruction. Consequently, patients experience a decrease in functional capacity and increasing disability [1]. The aim of pharmacotherapy in RA is to improve quality of life and function, and to prevent structural damage and, thus, improve long-term outcome of the disease. Many conventional synthetic disease-modifying anti-rheumatic drugs (csDMARDs) and biological disease-modifying anti-rheumatic drugs (bDMARDs) are now available [1–3]. Early and persistent systematic use of these treatment options, often as combination drug therapy, has dramatically reduced the severity and impact of RA, leading to substantial reduction in disease activity and joint damage [4–8].

The European League Against Rheumatism (EULAR) recommends that people with inflammatory arthritis should have access to patient education throughout the course of their disease [9]. The content and delivery of patient education should be individually tailored. Patients should be educated on their medication’s purpose, mode of action, possible side effects and monitoring guidelines [10]. Improving patients’ ability to make informed choices and to use medication effectively and safely should result in significant benefits for the health service and improve patient well-being [11].

Our institute’s yearly Patient Reported Experience Measure (PREM) on the quality of care that is being delivered; the Consumer Quality Index (CQI), revealed that the current level of our medication education needs some improvement. At the end of the first quarter of 2018, 34% of the patients answered in the negative to the question: ‘Did you received information about the side effects of your medication’. However, further details were missing, as the CQI results does not specify topics on which patients might have unmet needs for information.

The aims of the current study were, therefore, to 1. Directly observe in daily clinical practice how rheumatologists and rheumatology nurses actually provide information on medication side effects to RA patients and 2. Estimate what proportion of RA patients still lack relevant information on medication side effects and in which specific domains information is missing.

## Materials and methods

### Study design

The CHERRIES checklist for reporting the results of internet surveys was followed [12]. A mixed methods design was used. Phase 1 is the observational phase in which rheumatology health care professionals (HCPs) were directly observed in daily clinical practice on how they provide information on medication side effects to RA patients; while, phase 2 is the assessment phase in which RA patients were questioned on their satisfaction with the information they had received on medication.

### Study setting

Phase 1 and phase 2 of the study were conducted at the Department of Rheumatology and Clinical Immunology at Medisch Spectrum Twente, a large teaching hospital in Enschede, The Netherlands, from December 2018 to April 2019.

#### Phase 1: observation of rheumatology healthcare providers

For the observational phase of the study, the hospital’s digital consultation schedule was prospectively screened for prescheduled visits by patients diagnosed with rheumatoid arthritis (diagnosis treatment code [DBC] 101) and the availability of one of the three rheumatology nurses who acted as independent observers. A total of six consultation hours were selected for observation. All observed HCPs and RA patients were blinded to the aim of the observations. The HCPs were informed that structured observations of consultation hours would be performed to ‘improve overall quality of care’ and gave verbal informed consent. Before the scheduled consultation, verbal informed consent was also obtained from the patients without detailing the exact purpose of the observations, i.e., information about medication side effects. In total, 12 blinded structured observations were performed during the regular consultation visits of patients diagnosed with RA. Six HCPs were observed: 4 rheumatologists, 1 rheumatology fellow and 1 rheumatology nurse. A structured case record form specifically designed for this study was used to measure whether the topics ‘medication’ and ‘medication side effects’ were discussed during each consultation. The case record form further contained the domains ‘method of proving the medication information’,’describing the content of information that was provided in the electronic patient file’ and ‘interaction with other medication’(Supplementary Appendix A).

#### Phase 2: assessment phase

In January 2019, the hospital’s digital information system was used to make a retrospective selection of patients with a diagnosis treatment code of rheumatoid arthritis (DBC 101) who had visited the rheumatology outpatient department in the previous two months. The electronic patient files of these patients were screened for those patients that had started or changed their medication over the six months prior to the last consultation. A convenience sample of the first 100 patients who met these inclusion criteria were sent a letter asking them to fill out and return an enclosed questionnaire. This questionnaire was an adaptation of the Dutch validated version of the Satisfaction with Information about Medicines Scale (SIMS) [13–15]. The SIMS questionnaire consists of 17 items, each referring to a particular aspect of information received about medication; for example, information on ‘what your medicine is for’ or ‘whether the medicine has any unwanted effects (side effects)’. For each item, respondents are asked to rate the amount of information they have received as: too much, about right, too little, none received or none needed. The responses can be analyzed at different levels. First, at individual item level, to provide an overview of types of information that an individual patient is lacking. Second, at item sum score level, to provide a total satisfaction rating. Each item is given a score of 1 when rated satisfactory, i.e., ‘about right’ or ‘none needed’ or is given a score of 0 when rated unsatisfactory, i.e., ‘too much’, ‘too little’ or ‘none received’. Sum scores range from 0 to 17 with higher scores indicating a higher degree of overall satisfaction with the amount of medication information received. Third, at two subscale score levels, to identify patients’ satisfaction with information about ‘the action and usage of medication’ (items 1–9) and with information about ‘the potential problems of medication’ (items 10–17) [13–15]. The Dutch SIMS questionnaire was adapted for the current study by adding several open questions on specific patient characteristics (Supplementary Appendix B). The entire questionnaire consisted of 25 items on two pages. None of the questions were mandatory. No incentives were offered.

### Ethical considerations

According to the Dutch Medical Research Involving Human Subjects Act, the observation and assessment survey studies did not need formal approval of a medical ethical review board, as they were observational nonintervention studies without a high burden to patients. However, in both phases, all patients were informed about the purpose of the study, who the investigator was and the voluntary nature of participation.

### Statistical analysis

All available data were analyzed. A formal sample size calculation was not performed. Descriptive statistics are presented as mean ± SD when continuous and normally distributed and medians with IQR when continuous and non-normally distributed. Categorical variables are presented as numbers with percentages. All statistical analyses were performed with IBM SPSS Statistics for Windows version 15.

## Results

### Observation phase

Three rheumatology nurses performed 12 blinded structured observations of 6 HCPs during their regular consultations with patients diagnosed with RA. The HCPs were 50 (± 10.8) years of age with 14 (± 10.7) years of practice, 67% were females. All observed patients were currently using medication for their RA. Patient characteristics and the types of medication used are shown in Table 1.

**Table 1:** Patient characteristics and types of medication used in observation and assessment phase

Patient characteristics	Phase 1: observation phase (N12)	Phase 2: assessment phase (N61)
Age	62.5 (± 10.2)	67 (± 10.3)
Gender	75% female	72% female
Disease duration	13 years	7 years
Education level		
No education		1 (2%)
Primary school		2 (3%)
Lower or preparatory vocational education		17 (28%)
Intermediate general secondary		11 (18%)
Intermediate vocational education		13 (21%)
Higher general secondary education		3 (5%)
Higher vocational education		13 (21%)
University		0
Other		1 (2%)
Not completed		3 (5%)
csDMARDS	10 (83%)	59 (97%)
Sulfasalazine	3 (25%)	8 (13%)
Methotrexate	4 (42%)	29 (47%)
Hydroxychloroquine	2 (17%)	7 (11%)
Leflunomide	1 (8%)	0
Plaquenil	0	15 (25%)
bDMARDS	3 (25%)	13 (21%)
Etanercept	2 (17%)	6 (10%)
Sarilumab	0	1 (2%)
Tocilizumab	0	3 (5%)
Adalimumab	0	1 (2%)
Infliximab	0	1 (2%)
Rituximab	1 (8%)	1 (2%)
NSAIDs	4 (33%)	3 (5%)
Diclofenac	1 (8%)	0
Naproxen	2 (17%)	2 (3%)
Brufen	1 (17%)	1 (2%)
Corticosteroids	3 (33%)	18 (30%)
Prednisolone	2 (17%)	17(28%)
Triamcinolone	1 (8%)	1 (2%)

New medication was started during 3/12 (25%) of the consultations and medication was changed during 4/12 (33%) consultations. The topic ‘medication’ was discussed during all observed consultations by all rheumatology healthcare providers; however, non-rheumatic co-medication was discussed in only 6/12 (50%) of the observed consultations.

When new medication was started, the purpose of this medication was explained in all cases (100%), as was its mode of action (100%). Particulars of its use in combination with other medication were discussed in 67%, but (possible) side effects were explained in only 33% of the cases.

When medication was changed, the expected effect of this change was discussed in 75% of cases, its use in combination with other medication was discussed in only 25%, but (possible) side effects were explained in 75% of the cases.

Overall, the topic ‘medication side effects’ was discussed during 7/12 (58%) of the observed consultations. One purpose of the observations was to study the methods HCPs use to address the topic ‘medication side effects’. In 5/12 (42%) of the observed consultations, the HCPs enquired in a closed-ended format (yes/no) whether the patient experienced medication side effects. During 2/12 (17%) consultations, the HCP used examples of the most common medication side effects in a closed-ended format (yes/no) to check whether the patient experienced these side effects. In 2/12 (17%) observations, the patient spontaneously mentioned the occurrence of possible medication side effects.

The method by which education was given on medication and medication side effects was verbal in 11/12 (92%), leaflet hand-out in 1/12 (8%), referral to website or video in 0/12 (0%) and referral to a specialized nurse in 1/12 (8%). One patient received both verbal information and a leaflet hand-out. However, the method and content of the medication education that was given during the consultations were not documented in the electronic patients file in any of the observations.

### Assessment phase

Sixty-one out of the 100 RA patients (61%) returned the questionnaire they had been sent. The questionnaire was fully completed by 56/61 (92%) of the responders.

The patient characteristics as well as the types of medication used by the responders are shown in Table 1. There were no significant differences in patient characteristics between responders and non-responders, data not shown.

Patients used medication in tablet form in 51/61 (84%), subcutaneous injections 21/61 (34%), intravenous 3/61 (5%) or other 1/61 (2%).

The method of medication education was reported to have been verbal by 54/61 (89%) of the responders, a medication leaflet hand-out by 22/61 (36%), referral to a specialized nurse 17/61 (28%), a website 2/61 (3%), referral to instruction video 1/61 (2%), or other 3/61 (5%). Furthermore, 34/61 (56%) reported that more than one method of medication education had been used.

**Fig. 1:**
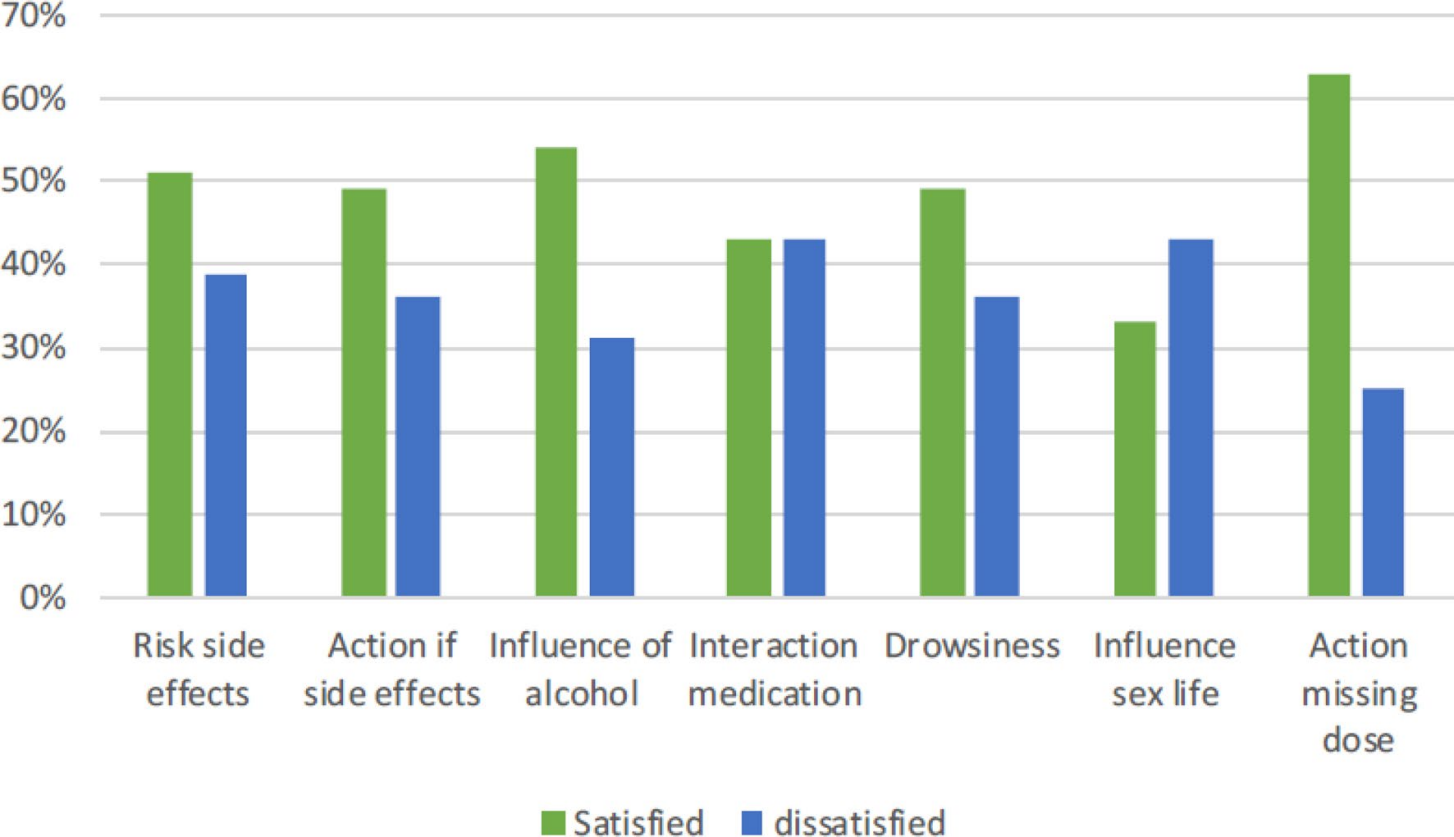
Topics on which responders have an unmet need for information

When asked about their overall satisfaction with their medication education, responders scored mean 7.3 (± 1.,9) on a numeric rating scale (range 0–10). The Satisfaction with Information about Medicines Scale (SIMS) showed a comparable total satisfaction rating sum score of mean 12.3 (± 4.4) (range 0–17), indicating a high degree of overall satisfaction with the amount of medication information received. Overall, 37/55 (67%) responders were satisfied with their medication education. At subscale score levels, 49/55 (89%) responders were satisfied with the amount of information about ‘the action and usage of medication’ (items 1–9), but only 26/55 (47%) responders were satisfied with the amount of information about ‘the potential problems of medication’ (item 10–17). An unmet need was identified when ≥ 25% of the responders rated the amount of information received on a specific topic as unsatisfactory; i.e., ‘too much’, ‘too little’ or ‘none received’. An unmet need for information was identified for the topics ‘What are the risks of you getting side effects’, ‘What you should do if you experience unwanted side effects’, ‘Whether you can drink alcohol whilst taking this medicine’, ‘Whether the medicine interferes with other medicines’, ‘Whether the medication will make you feel drowsy’, ‘Whether the medication will affect your sex life’, ‘What you should do if you forget to take a dose’, as shown in Fig. 1.

Finally, in an open text field, patients were asked to suggest possible ways the department of Rheumatology could improve their medication education. Suggestions mentioned were: ‘provide a written rendition of the information that was provided during the consultation so it can be read back’, ‘ask more often about medication side effects during the consultations or by another method, for example through the questionnaires that are completed every 3 months by all patients of the Rheumatology department’, ‘schedule a telephone consultation a few days after medication information was provided to verify whether the information was understood and to answer any questions’, ‘provide a written summary of what was discussed during the consultation so it can be read back’.

## Discussion

The results of the current study confirm the results of our hospital’s yearly Consumer Quality Index (CQI) questionnaire on the quality of care that is being delivered. In the 2018 CQI, 34% of the patients attending the department of rheumatology had answered negatively to the question: ‘Did you receive information about the side effects of your medication’. However, the CQI questionnaire does not further specify topics on which patients might have unmet needs for information. The current study shows that while rheumatoid arthritis (RA) patients actually had a high degree of overall satisfaction with the medication information they had received, they were particularly satisfied with the amount of information on the action and usage of medication but less so with the amount of information they had received on potential problems of the medication; such as the risks of having side effects, the impact of side effects on daily life, and what to do about side effects. This was perceived as an unmet need.

Another recent study from the Netherlands by Mathijssen et al. showed that RA patients have needs for informational, practical and emotional support with regard to medication use [16]. Informational support appeared to be most important. Informational support encompassed provision of information and facts, including advice, suggestions and feedback from healthcare providers [16].

Several studies confirm the results of the present study, showing that RA patients are generally satisfied with the information received. A recent study by Packham et al. on patient attitudes and experience of information received during drug counseling for RA medications showed that patients were generally very satisfied with the quantity, quality and timeliness of information received [17]. Furthermore, 95% of the responders stated having received information that they did not know before. Drug information received from rheumatology nurses, rheumatology doctors and information leaflets were found to be most useful. Strikingly, only 40% of the participants reported previously having been aware of important drug side effects [17]. However, the current study further demonstrates that despite general satisfaction with medication education, there might still be unmet needs for specific information, specifically on potential risks and side effects.

Whether medication education actually influences medication adherence in RA patients was studied by Taibanguay et al. [18]. Patient education was indeed shown to significantly improve medication adherence. Use of a disease information leaflets, both with or without direct counseling, was equally associated with improved medication adherence in patients with RA [18].

A study by Geryk et al. confirmed that greater receipt of information was associated with greater medication adherence, use of more medications and with more satisfaction with doctor medication-related support, but conversely also with greater medication-taking concerns [19].

In the current study, no clear preferred method of medication education was used. Our department encourages rheumatology healthcare professionals to distribute written medication information, i.e., medication leaflet hand-outs, to supply standardized high-quality information in clear language, which can be read, re-read and discussed and which is available in several different languages. Noticeably, 88.5% of responders reported having received verbal medication information, while only 36% reported having been handed the medication leaflet. Whether this lack of adherence to department protocol was due to time constraints, lack of availability of materials, recall bias, or a result of tailoring to individual patients’ needs was not investigated in this study. Currently, specific high-quality medication information is increasingly becoming available online, either as website texts, illustrations or as video content. The latter often also specifically designed for patients with functional illiteracy. However, the objectivity and partiality of much of this content remain a concern.

The Satisfaction with Information about Medicines Scale (SIMS) as used in this study can also be systematically applied in daily clinical practice [13–15]. At aggregate sum score and subscale levels, the questionnaire may be used to monitor overall satisfaction with the amount of medication information received by specific groups of patients. Furthermore, at item level, the questionnaire can be used to identify and prioritize types of medication information that an individual patient is lacking. This detailed information may subsequently be used to guide healthcare providers when providing medication education to that specific patient.

Finally, the current study showed that caregivers consequently failed to document the method and content of the medication information that was provided. Besides this being a legal obligation, by not clearly documenting the type and amount of medication information that is provided, it is difficult for subsequent caregivers to anticipate the needs and uncertainties of patients.

### Strength and limitation

A strength of the current study was that both structured observation of actual rheumatology consultations and a questionnaire-based assessment of patients’ experiences and needs were conducted to asses all aspects of RA medication education. Furthermore, the RA-patient population was representative of that regularly seen at outpatient rheumatology departments, being mostly female, mean 67 years of age, with intermediate vocational education or apprenticeship training and mostly using conventional synthetic DMARDs.

A possible limitation of the current study is that only one rheumatology department was evaluated cross-sectionally in which a total of six consultation hours were observed, making it difficult to draw any definite conclusions. It is possible that local practice may deviate from common practice or may change over time. However, the current results appear largely in accordance with those published in the literature. Another possible limitation was that different patients participated in the observation phase and the assessment phase. Also, albeit relatively high, a questionnaire response rate of 61% may still cause selection bias. Furthermore, not all returned questionnaires had been entirely completed. As with all retrospective questionnaires the possibility of recall bias and response bias exists. However, because the response was anonymous and the sample size relatively large, we feel that these effects are unlikely to have strongly influenced the results of this study. The length of time between starting or changing medication and filling out the questionnaire may also have varied, causing further possibility of recall bias.

## Conclusion

Rheumatoid arthritis patients express overall high satisfaction with their medication education. However, there is still an unmet need for information on potential risks and side effects. Specific topics on which patients lack medication information were: influence on sex life, influence of alcohol, interaction with other medication, risk of side effects, drowsiness, what to do about side effects and what to do when doses are missed. Using the SIMS questionnaire in daily clinical practice may help focus medication education to the needs of the individual patient.

## Supplementary Information

Below is the link to the electronic supplementary material.

Supplementary file1 (DOCX 17 KB)

Supplementary file2 (DOCX 32 KB)

Supplementary file3 (DOCX 137 KB)

## Declarations

### Availability of data and material

All data included in this study are available upon reasonable request by contact with the first corresponding author.
