# Supplementary material for: Patient perspectives on how to improve education on medication side effects: cross-sectional observational study at a rheumatology clinic in The Netherlands
Source: Rheumatol Int. 2021 Mar 17;41(5):973–9. doi: 10.1007/s00296-021-04815-5 (PMC8019410; doi:10.1007/s00296-021-04815-5)
Supplement: Supplementary file 1 — Supplementary file1 (DOCX 17 KB) [file 296_2021_4815_MOESM1_ESM.docx]

| Pt id number: | |  |  |  |  |  |  |  |  |  |  |  |  |
| --- | --- | --- | --- | --- | --- | --- | --- | --- | --- | --- | --- | --- | --- |
|  |  |  |  |  |  |  | Yes | No | Name rheumatology healthcare provider | | | | |
| Pt allready uses medication | | |  |  |  |  |  |  |  |  |  |  |  |
|  |  |  |  |  |  |  |  |  |  |  |  |  |  |
| The topic 'medication' was discussed during consultation | | | | | |  |  |  | Current use anti TNF / biological yes/no | | | | |
|  |  |  |  |  |  |  |  |  |  |  |  |  |  |
| New medication | |  |  |  |  |  |  |  |  |  |  |  |  |
| New medication was prescribed during consultation | | | | | |  |  |  |  |  |  |  |  |
| The purpose of this medication was explained | | | | |  |  |  |  |  |  |  |  |  |
| (possible) side effects were explained | | | |  |  |  |  |  |  |  |  |  |  |
| Mode of action was explained | | |  |  |  |  |  |  | Notes: |  |  |  |  |
| Use with other medication was discussed | | | | |  |  |  |  |  |  |  |  |  |
|  |  |  |  |  |  |  |  |  |  |  |  |  |  |
| Current medication | |  |  |  |  |  |  |  |  |  |  |  |  |
| Healthcare provider asked whether pt experienced side effects yes/no | | | | | | |  |  |  |  |  |  |  |
| Side effects where checked on the base of examples | | | | | |  |  |  |  |  |  |  |  |
| Pat reported (possible) side effects | | | |  |  |  |  |  |  |  |  |  |  |
|  |  |  |  |  |  |  |  |  |  |  |  |  |  |
| Change of medication | | |  |  |  |  |  |  |  |  |  |  |  |
| Current medication was changed during consultation | | | | | |  |  |  |  |  |  |  |  |
| (possible) side effects were explained | | | |  |  |  |  |  |  |  |  |  |  |
| The expected effect of this change was discussed |  |  |  |  |  |  |  |  |  |  |  |  |  |
| Interaction with other medication was discussed | | | | |  |  |  |  |  |  |  |  |  |
|  |  |  |  |  |  |  |  |  |  |  |  |  |  |
| Method of communication | | |  |  |  |  |  |  |  |  |  |  |  |
| Verbal |  |  |  |  |  |  |  |  |  |  |  |  |  |
| Leaflet hand-out | |  |  |  |  |  |  |  |  |  |  |  |  |
| Referral to website / instruction video | | | |  |  |  |  |  |  |  |  |  |  |
| Referral to specialized nurse | | |  |  |  |  |  |  |  |  |  |  |  |
|  |  |  |  |  |  |  |  |  |  |  |  |  |  |
| Description | |  |  |  |  |  |  |  |  |  |  |  |  |
| Note has been made in the electronic patient file | | | | |  |  |  |  |  |  |  |  |  |
| concerning medication education | | | |  |  |  |  |  |  |  |  |  |  |
