# Supplementary material for: Patient perspectives on how to improve education on medication side effects: cross-sectional observational study at a rheumatology clinic in The Netherlands
Source: Rheumatol Int. 2021 Mar 17;41(5):973–9. doi: 10.1007/s00296-021-04815-5 (PMC8019410; doi:10.1007/s00296-021-04815-5)
Supplement: Supplementary file 2 — Supplementary file2 (DOCX 32 KB) [file 296_2021_4815_MOESM2_ESM.docx]

We would like to ask you about the information you have received about your medicines for your rheumatic disease. Please rate the information you have received about each of the following aspects of your medicines. If you use more than one medicine for your rheumatic disease, please give your overall feeling about information you have received about all your medicines for your rheumatic disease.

|  | Too much | About right | Too little | None received | None needed |
| --- | --- | --- | --- | --- | --- |
| What your medicine is called |  |  |  |  |  |
| What your medicine is for |  |  |  |  |  |
| What it does |  |  |  |  |  |
| How it works |  |  |  |  |  |
| How long it will take to act |  |  |  |  |  |
| How you can tell if it is working |  |  |  |  |  |
| How long you will need to be on your medicine |  |  |  |  |  |
| How to use your medicine |  |  |  |  |  |
| How to get a further supply |  |  |  |  |  |
| Whether the medicine has any unwanted effects (side effects) |  |  |  |  |  |
| What are the risks of you getting side effects |  |  |  |  |  |
| What you should do if you experience unwanted side effects |  |  |  |  |  |
| Whether you can drink alcohol whilst taking this medicine |  |  |  |  |  |
| Whether the medicine interferes with other medicines |  |  |  |  |  |
| Whether the medication will make you feel drowsy |  |  |  |  |  |
| Whether the medication will affect your sex life |  |  |  |  |  |
| What you should do if you forget to take a dose |  |  |  |  |  |

We would like to ask you to answer a few general questions to describe the patient group that uses medication for their rheumatic disease.

Gender:

- Male
- Female

Age:

……………… years

Highest completed education (completed with a diploma)

- No education (primary school: unfinished)
- Primary school
- Lower or preparatory vocational education (e.g. LTS, LEAO, LHNO, VMBO)
- Intermediate general secondary education (e.g. MAVO, (M)ULO, MBO-kort, VMBO-t)
- Intermediate vocational education and apprenticeship training (e.g. MBO-lang, MTS, MEAO, BOL, BBL, INAS)
- Higher general secondary education and pre-university education (e.g. HAVO, VWO, Atheneum, Gymnasium, HBS, MMS)
- Higher vocational education (e.g. HBO, HTS, HEAO, HBO-V)
- University

|  |
| --- |

- Other:

**Method of education:**

Which method was used to inform you about your medication for your rheumatic disease:

- Verbal
- Medication leaflet
- Instruction by rheumatology nurse at the department of Rheumatology
- Instruction video
- Directed to a website

|  |
| --- |

- Other:

**Kind of medication:**

What is the name of the medication that you use for your rheumatic disease at this moment:

|  |
| --- |

**Method of use of medication:**

Which method is used for the medication for your rheumatic disease (several options are possible)

- Tablets
- Injection
- Intravenous

|  |
| --- |

- Other

**Satisfaction with medication education:**
How satisfied are you concerning the medication education for the medication for your rheumatic disease


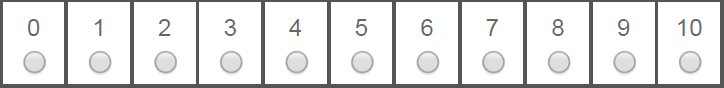


Very Very
Dissatisfied satisfied

**Ways of improvement:**

How can the department of Rheumatology improve their medication education:

|  |
| --- |

**Thank you very much for completing this questionnaire!**
