# Supplementary material for: Patient perspectives on how to improve education on medication side effects: cross-sectional observational study at a rheumatology clinic in The Netherlands
Source: Rheumatol Int. 2021 Mar 17;41(5):973–9. doi: 10.1007/s00296-021-04815-5 (PMC8019410; doi:10.1007/s00296-021-04815-5)
Supplement: Supplementary file 3 — Supplementary file3 (DOCX 137 KB) [file 296_2021_4815_MOESM3_ESM.docx]

Assessment phase

Observational phase

Double blinded observations healthcare professionals

(n=12)

By RA patients completed questionnaire based on SIMS

(n=61)

**RESULTS**

**RESULTS**


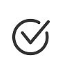


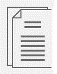

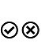

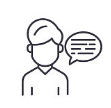

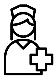
Medication side effects were discussed during consultation Methods of medication education

42%

28%

36%

89%

58%


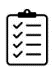


17%

*Closed-ended question*

*Medication leaflet hand-out*

*Refferal to a specialized nurse*

*Verbal*

*Example with closed-ended question*


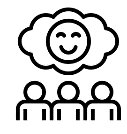


Overall satisfaction with their medication education.
Mean **7.3** on a numeric rating scale (range 0-10)

**DIRECTIONS FOR THE FUTURE**


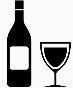

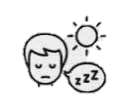

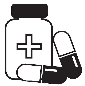

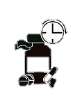

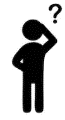

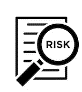

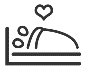

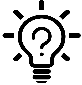

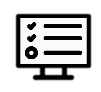

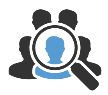


*The influence of alcohol*

31%

*Whether the medication can cause drowsiness*

36%

*Interaction with other medication*

43%

*What to do when you forget to take a dose*

25%

*What to do when getting side effects*

36%

*Risks of getting side effects*

39%

*Whether the medication can effect the sex life*

43%

Unmet need for information

**Using the SIMS questionnaire in daily clinical practice may help focus medication education to the needs of the individual patient**

1. To observe how rheumatology HCPs deliver medication information (observational phase)

2. To determine in which specific domains information is missing (assessment phase)

Objectives
